# Optimizing the Face Paradigm of BCI System by Modified Mismatch Negative Paradigm

**DOI:** 10.3389/fnins.2016.00444

**Published:** 2016-10-07

**Authors:** Sijie Zhou, Jing Jin, Ian Daly, Xingyu Wang, Andrzej Cichocki

**Affiliations:** ^1^Key Laboratory of Advanced Control and Optimization for Chemical Processes, Ministry of Education, East China University of Science and TechnologyShanghai, China; ^2^Brain Embodiment Lab, School of Systems Engineering, University of ReadingReading, UK; ^3^Laboratory for Advanced Brain Signal Processing, Brain Science Institute, RIKENWako-shi, Japan; ^4^Systems Research Institute PAS, Warsaw and Nicolaus Copernicus University (UMK)Torun, Poland

**Keywords:** brain computer interface, P300, N400, face paradigm, online system

## Abstract

Many recent studies have focused on improving the performance of event-related potential (ERP) based brain computer interfaces (BCIs). The use of a face pattern has been shown to obtain high classification accuracies and information transfer rates (ITRs) by evoking discriminative ERPs (N200 and N400) in addition to P300 potentials. Recently, it has been proved that the performance of traditional P300-based BCIs could be improved through a modification of the mismatch pattern. In this paper, a mismatch inverted face pattern (MIF-pattern) was presented to improve the performance of the inverted face pattern (IF-pattern), one of the state of the art patterns used in visual-based BCI systems. Ten subjects attended in this experiment. The result showed that the mismatch inverted face pattern could evoke significantly larger vertex positive potentials (*p* < 0.05) and N400s (*p* < 0.05) compared to the inverted face pattern. The classification accuracy (mean accuracy is 99.58%) and ITRs (mean bit rate is 27.88 bit/min) of the mismatch inverted face pattern was significantly higher than that of the inverted face pattern (*p* < 0.05).

## Introduction

Brain-computer interfaces (BCIs) are intended to help patients to communicate with other people or control external devices through their brain activity (Wolpaw et al., 2000b; He et al., 2013). Patients who suffer from Amyotrophic lateral stenosis (ALS) could be helped by this technology. Scalp electroencephalography (EEG) is convenient in experimental setups. Therefore, it is widely used and studied (Mak et al., 2011). Event-related potential (ERP) based BCI systems are amongst the most commonly used BCIs and are often used for designing speller systems (Hwang et al., 2013; Zhang D. et al., 2013).

The first P300-based speller system was presented and used a 6 × 6 stimuli matrix with 36 targets (Farwell and Donchin, 1988). The study showed the potential value of ERP-based BCIs for designing speller systems. However, to overcome problems with the signal to noise ratio of the ERP it was necessary to base control upon the construction of averaged trials, which decreased the information transfer rates (ITRs) of the system (Wolpaw et al., 2002). Therefore, many studies have focused on improving the performance of ERP-based BCIs in practical applications (Sellers et al., 2006). Optimized classifiers were presented to improve the classification accuracy when only a few trials were used for constructing the average ERP (Zhang Y. et al., 2013). Generic models, using online training methods, were presented to decrease the offline calibration time (Lu et al., 2009; Jin et al., 2013; Tobias et al., 2013). In addition to the mathematic methods, the paradigms used to evoke ERPs and stimulus patterns were also studied to enhance the difference between target and non-target trials. Martens et al. presented several P300-based BCI systems using different target-to-target intervals (TTIs) to show the refractory and overlap effects in ERPs (Martens et al., 2009). Some work optimized the sequence of stimuli to avoid double flashes, which decreased the repetition blindness and increased the classification accuracy and ITRs (Jin et al., 2011b; Townsend et al., 2012). Hong et al. reported that motion onset potentials (the N200) evoked by moving targets could be used to improve the performance of ERP-based BCIs (Hong et al., 2009). A paradigm was designed to evoke both N200 and P300 to improve the discrimination of target and non-target trials (Jin et al., 2012). Finally, hybrid systems have also been proposed. For example, Long et al. designed a BCI system using P300 and motor imagery for multi-degree control of a wheelchair (Long et al., 2012) and Yin et al. combined P300 and steady-state visually evoked potential (SSVEP) brain signals for a high-performance BCI-based speller system (Yin et al., 2013, 2014, 2015).

Kaufmannn et al. first used images of faces as stimuli, and showed that they could be used to obtain high classification accuracies with both healthy and disabled BCI users with high ITRs (Kaufmann et al., 2011, 2013). The facial expression change paradigm has also been demonstrated to evoke discriminative ERPs and obtained equally high classification accuracies as the face pattern (Jin et al., 2014b).

It was reported that the inverted face pattern could evoke a large N170, a vertex positive potential (VPP), and yield better performance than the face pattern (Zhang et al., 2012). In their work, seven volunteers participated in the experiment in which eight targets were presented on screen, and nine patterns were compared: an upright face, inverted face, upright eyeless face, inverted eyeless face, upright eye, inverted eye, upright object, inverted object, and highlight icon. The online ITR of the invert face pattern were the best among them and had significantly longer latencies before the N170 and VPP. The N170 and VPP are sensitive to configural processing (Gruss et al., 2012).

It was reported that traditional P300-based BCIs could be improved significantly through a modification of the mismatch paradigm (Jin et al., 2014a, 2015). Ten subjects were paid to participate in the experiment. The character “D” was the deviant stimulus and the character “S” was the standard stimulus. The modified mismatch paradigm could evoke significantly larger N200 and N400 ERPs compared to the traditional P300 paradigm. Based on previous studies, inverted and upright face images with different expressions were used to design the mismatch inverted face pattern (MIF-pattern). The visual stimulus modality elicits a visual mismatch negativity (vMMN). Consistent with the auditory MMN, the vMMN elicits an N200 (Kimura et al., 2008, 2010a). The vMMN with emotional faces can also elicit an N400 (Bobes et al., 2000).

The face stimuli contained more configural information than the character stimuli. A natural question is whether the invited face pattern could be used in the vMMN pattern. This study tries to explore the possibility of improving the vMMN pattern by combining it with the inverted face pattern. Our hypotheses are that the mismatch negative face pattern (MIF-pattern) will evoke significantly larger N200 and N400 amplitudes and that this could be used to obtain significantly higher classification accuracies and ITRs compared to the inverted face pattern (IF-pattern). In this paper, second section is the Methods and Materials, which introduce the experiment and method used in this study. Third section is the Result which shows the performance of the presented paradigm. Fourth section is the Discussion and fifth section is the Conclusion.

## Methods and materials

### Participants

Ten healthy participants (7 male and 3 female, aged 23–25, mean 23.6) were paid for participated in the study. Four participants did not have any experience with BCI. All the participants were asked to remain relaxed during data acquisition. All subjects were informed and signed a written consent form prior to this experiment, and were paid 50 RMB for their participation in each session.

**Figure 1:**
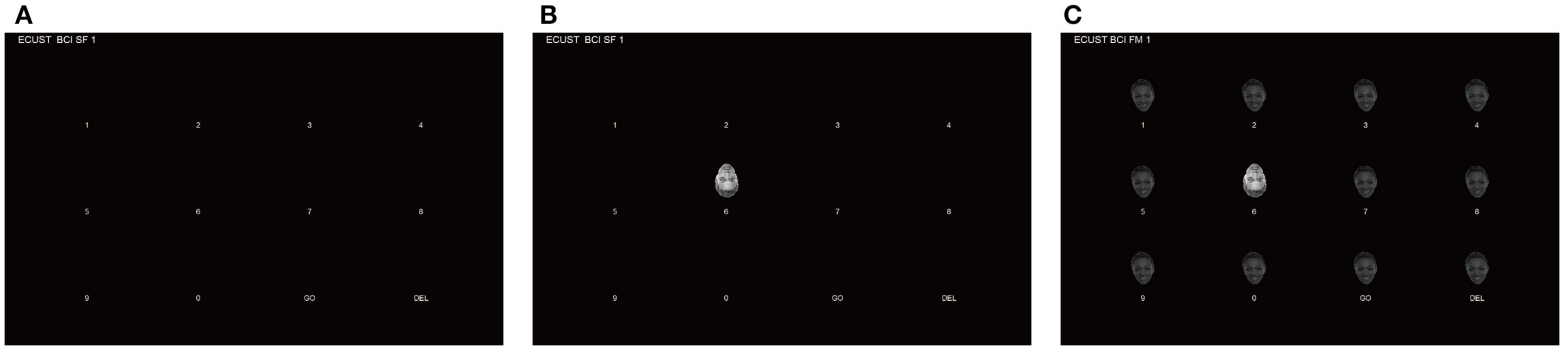
**The interface that was shown to the participants**. **(A)** The matrix without stimuli. **(B)** An example of the stimulus in the IF-pattern. **(C)** An example of the stimulus in the MIF-pattern. The feedback appeared at the top of the screen during online sessions.

**Figure 2:**
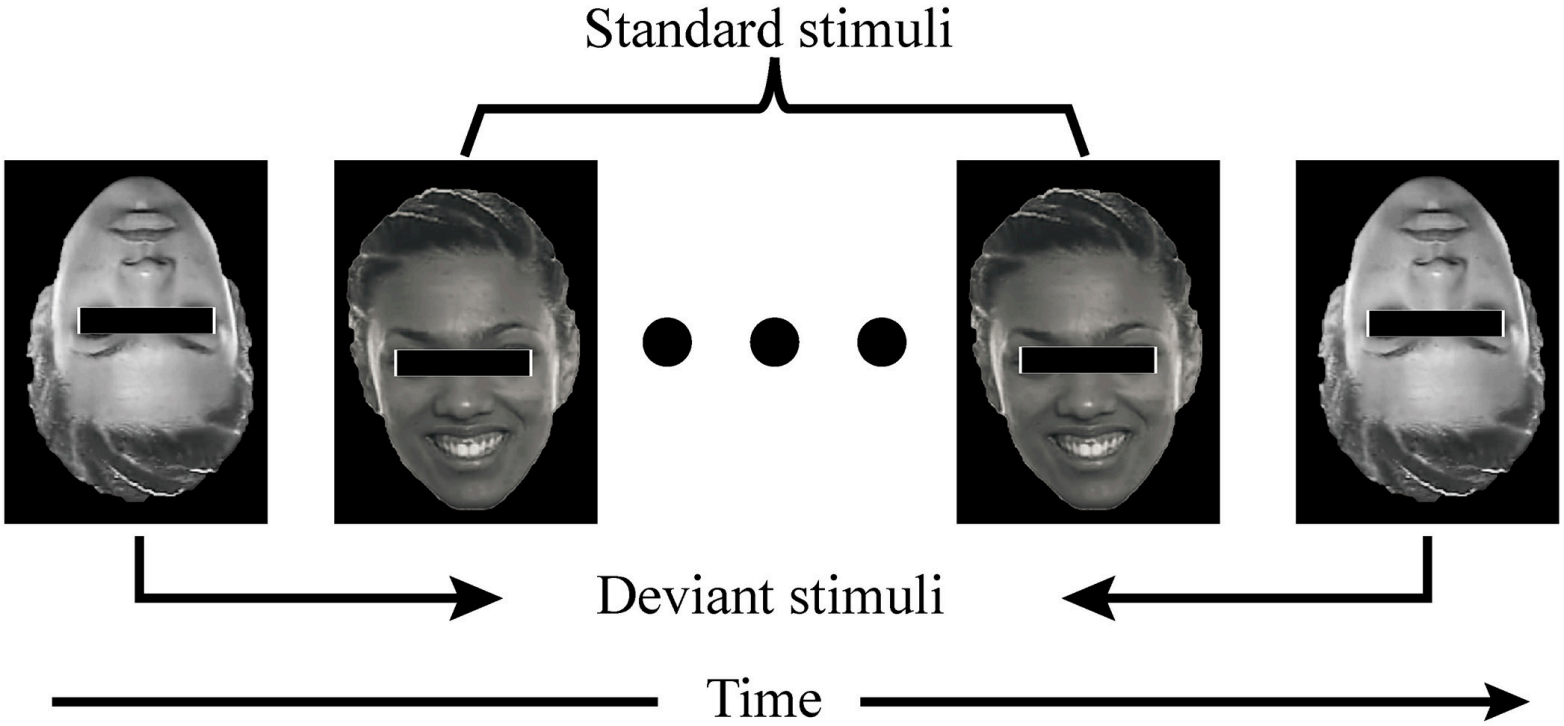
**The figure demonstrates what participants saw in one item**. There were several standard stimuli (at least six) between two deviant stimuli.

The participants were seated ~85 cm in the front of a computer monitor, which was 30 cm long by 48 m wide. The display presented to the participants is shown in Figures 1, 2. Twelve items were presented in a 3 × 4 arrangement. The participants' task was to focus their attention on the desired item in the matrix and count the number of times the face appeared directly above the item. The participants were trained simply to guide them as to how to do the tasks before they began the experiment.

### Experiment setup, Off-, and online protocols

**Figure 3:**
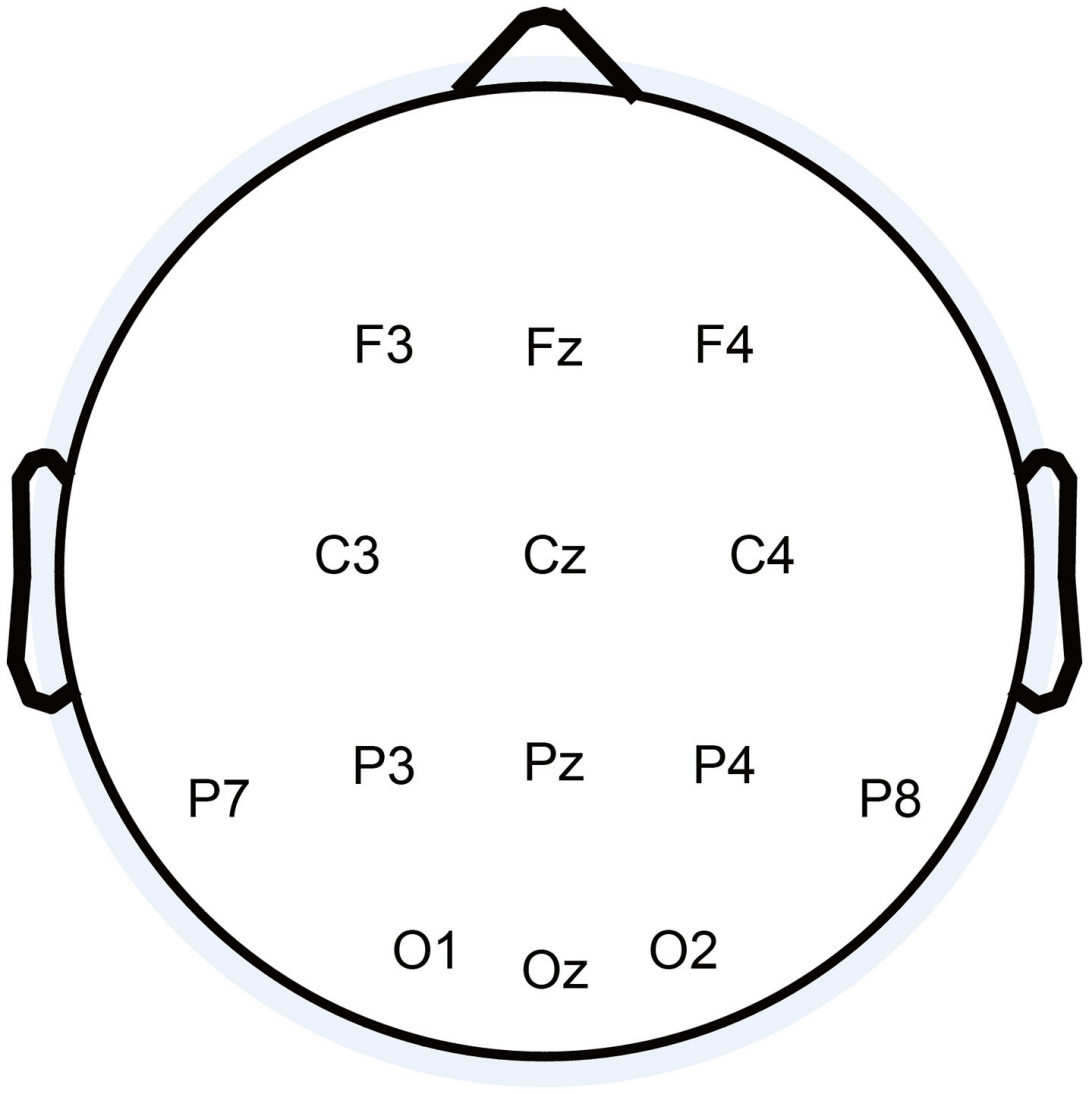
**The 16 electrodes used in this study**.

EEG signals were recorded with a g.HIamp and a g.EEGcap (Guger Technologies, Graz, Austria), band pass filtered between 0.1 and 60 Hz, notch filtered at 50 Hz and sampled at 512 Hz. Fourteen active electrodes were used, as shown in Figure 3. The right mastoid electrode served as the reference, and the ground electrode was placed on the forehead (FPz).

A female face (actress Freema Agyeman) was selected for use in this study, the face figures were obtained from a video of emotional research. Faces with negative and positive valence were used to evoke ERPs. In the IF-pattern, the inverted face with negative valence (i.e., the deviant stimulus), was presented above one of the 12 items (Figure 1B). The MIF-pattern was similar to the IF-pattern, with one exception. When the inverted face appeared above one of the items, gray faces with positive emotions (the standard stimulus) appeared above other 11 items (Figure 1C). In other words, the background of the stimulus was the flicking gray faces in the MIF-pattern. The gray face was replaced by an inverted woman's face, which was used as the stimulus in the MIF-pattern (see Figure 2). Several standard stimuli (flicking gray faces) appeared before the deviant stimuli (an inverted woman's face), thereby producing a “visual mismatch.” The inter-stimulus-interval (ISI) of the stimulus was 100 ms and the stimulus onset asynchrony (SOA) was 300 ms in both patterns, which was the same for the flicking background used in the MIF-pattern.

It was reported that there should be more than four standard stimuli before the deviant stimuli to evoke clear mismatch negative potentials (Kimura et al., 2006). One of the stimuli was shown in Figure 2. Items 1–6 were in group 1, the others 6 stimuli (items 7–12) were in group 2. The stimuli order of items 1–6 was randomized and the order of items 7–12 was also randomized. The strategy used was to flash the items in group 1 randomly and then flash the items in group 2 randomly in each trial. All the 12 items in group 1 and 2 were flashed once in each trial. In this way, only a subset of items in group 1 in the first trial did not meet the standard stimulus number before the deviant stimulus was presented.

Participants had to complete two offline phases (3 offline runs per phase) for two patterns, after which there were two online runs. Each offline run contained 5 trial-blocks, that each contained 16 trials, and each online run contained 24 trial-blocks (the targets were items 1–12). The number of trials per trial-block was variable in the online phase, as described in Section Adaptive System Settings. Before one trial-block, a green box was briefly displayed to indicated the target item of the trial-block. A 2-min break was given to participants between two runs. The runs of patterns were presented in the same order as those in the offline phase, and the order was counterbalanced over participants. Participants completed all experiment stages in 2 h.

### Feature extraction procedure

The first 800 ms of EEG data after each stimulus presentation were used to extract features from the 14 EEG channels (shown in Figure 3). EEG data was filtered into the range 0.1–30 Hz using a third order Butterworth band pass filter. After filtering, the data were down-sampled from 512 to 73 Hz by selecting every seventh sample. So the size of the feature vector for one stimulus was 14 × 58 (14 channels by 58 time points).

### The information transfer rates (IRTs)

The bit rate is the measure of ITRs we used. Both speed and accuracy affect the bit rate (Wolpaw et al., 2000a). Bit rate is define as

(1) $$\begin{array}{rcl} \text{Br} & = & \left[\log_{2}\;N+P\;\log_{2}\;P\;+\;\left(1\;-\;P\right)\log_{2}\left(\frac{1-P}{N-1}\right)\right]\\ /\left(\frac{60}{T\;\times \;AVT}\right) \end{array}$$

where Br denotes the bit rate, *N* denotes the number of targets, and *P* denotes the accuracy. *T* denotes the time (in seconds) for a trial to complete. *AVT* denotes the number of trials used to construct the average used in each trial-block for each participant; In this study *N* is set to 12, *T* is set to 3.6 s (12 stimuli and the SOA is 0.3 s).

### Classification scheme

The classifier used in this study was a Bayesian Linear Discriminant Analysis (BLDA) classifier, which may be seen as an extension of Fisher's Linear Discriminant Analysis (FLDA; Penney et al., 2001; Hoffmann et al., 2008).

The classification rule is defined as,

(2) $$\begin{array}{r} \text{m}=\;\beta \;{\left(\beta \text{X}{\text{X}}^{T}+I^{{}^{\prime }}\left(\alpha \right)\right)}^{-1}\text{Xt} \end{array}$$

(3) $$\begin{array}{r} \text{y }=\;{\text{m}}^{\prime }\text{x} \end{array}$$

where m is the discriminant vector used for the classification, and y is the output of the classifier. X denotes the matrix, each column of which contains a feature vector, and t denotes the regression targets. The value of t is set to *N*/*N*_1_ for class 1, and −*N*/*N*_2_ for class −1 (where *N*_1_ and *N*_2_ are the number of features from class 1 and class −1, and *N* is the sum of *N*_1_ + *N*_2_). The two hyper parameters α and β are the inverse variance of prior distribution and noise. They can be determined with an iterative method.

Features acquired from offline data were used to train the classifier and resulted in the classifier model. The data sets used to train the classifier model contained 240 target stimuli and 2640 non-target stimuli per participant.

In the online phase, single trials were classified immediately after the data was completely acquired (800 ms after the onset of the last stimulus in a trial). The 12 classifier outputs, one per output per stimulus, were summed over trials. The stimulus with the maximum summed output was considered to be the target.

### Adaptive system settings

The number of trials per trial-block was automatically selected during the online runs (Jin et al., 2011a). In the online experiment, the system obtained a detected target by using the classifier after each trial. In the first trial, the stimulus whose features obtained the highest classifier score in the trial was regarded as the detected target. For subsequent trials, the classifier scores of the stimuli in that trial were averaged with the classifier scores of the corresponding stimuli in the previous trials. The highest averaged classifier score indicated the detected target of that trial. If the two successive detected stimuli were the same, the corresponding item was regarded as the correct target and shown on the top of the screen. Then, the participant could move on to attempt to select the next character.

## Results

### The online performance

Table 1 shows the online classification accuracy, bit rate, and average number of trials used per participant.

**Table 1:** **The classification accuracy, trials used to construct the average, and ITRs during online experiments**.

**Participants**	**Acc (%)**		**AVT**		**ITR (bit/min)**	
	**IF-P**	**MIF-P**	**IF-P**	**MIF-P**	**IF-P**	**MIF-P**
S1	95.83	100.0	2.17	2.29	24.55	26.07
S2	91.67	100.0	2.38	2.21	20.23	27.06
S3	95.83	100.0	2.33	2.08	22.79	28.68
S4	100.0	100.0	2.04	2.08	29.27	28.68
S5	100.0	100.0	2.21	2.13	27.06	28.12
S6	100.0	100.0	2.00	2.00	29.87	29.87
S7	100.0	100.0	2.00	2.00	29.87	29.87
S8	87.5	95.83	2.38	2.29	18.31	23.21
S9	100.0	100.0	2.17	2.08	27.58	28.68
S10	100.0	100.0	2.21	2.17	27.06	27.58
Avg	97.08	99.58	2.19	2.13	25.66	27.78

*Acc, classification accuracy; AVT, Trials for constructing the average used in each trial-block per participant; RBR, raw bit rate; IF-P, IF-pattern; MIF-P, MIF-pattern*.

These data were statistically tested for normality (One-Sample Kolmogorov Smirnov test) and sphericity (Mauchly's test). Since the classification accuracy was not normally distributed, a non-parametric Kendall test was used to test the differences in classification accuracies between the SF and MIF patterns. The classification accuracy of the MIF-pattern was significantly higher than that of the IF-pattern (*p* < 0.05). Paired samples *t*-tests were used to test the differences between the MIF- and IF-patterns in terms of bit rates. The ITRs (Wolpaw et al., 2002) of the MIF-pattern were significantly higher than that of the IF-pattern (*t* = −2.7, *p* < 0.05).

### ERP

**Figure 4:**
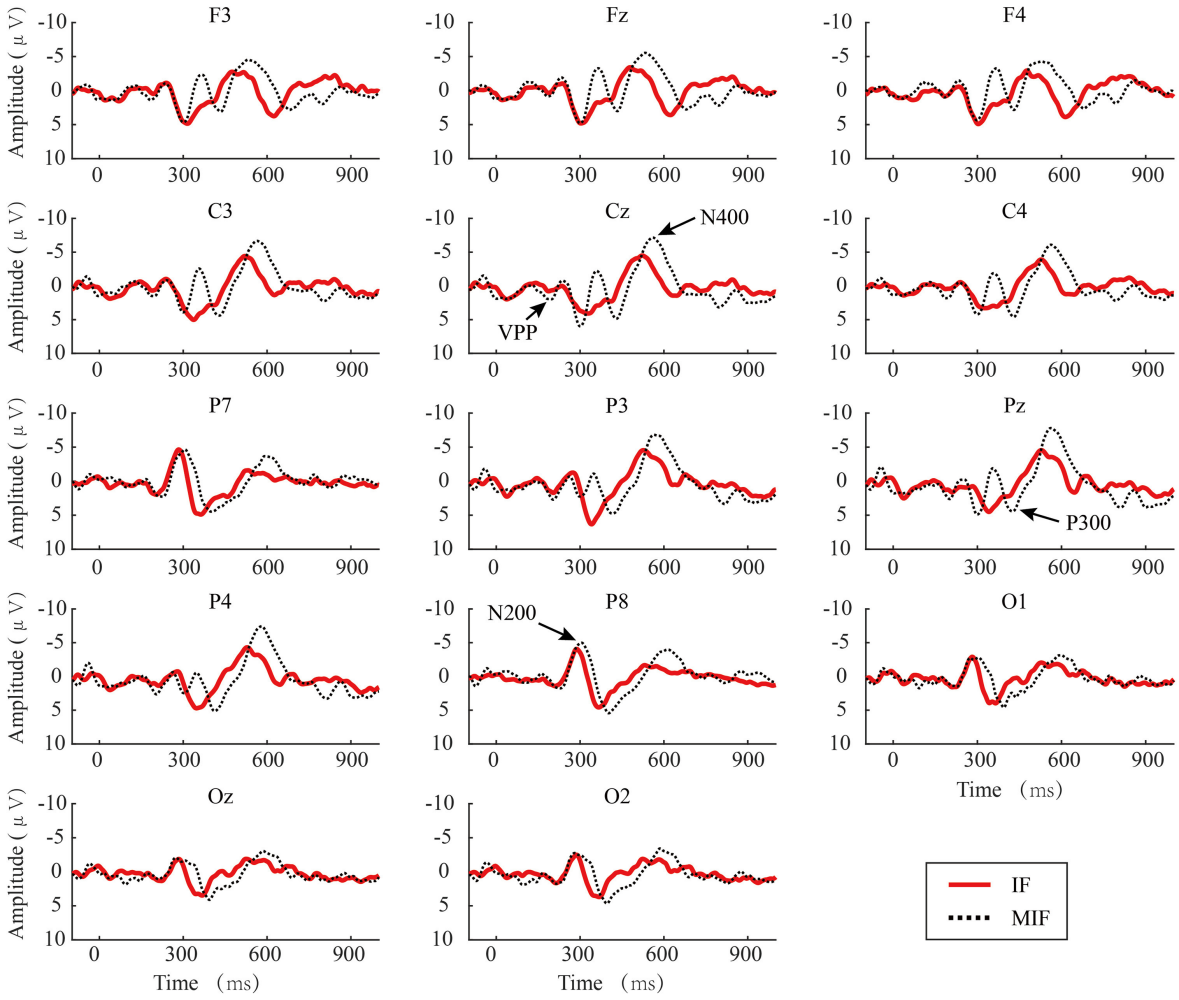
**The grand average ERPs of deviant stimuli for the IF and MIF patterns**. Four ERPs recorded during presentation of the MIF-pattern are displayed.

**Figure 5:**
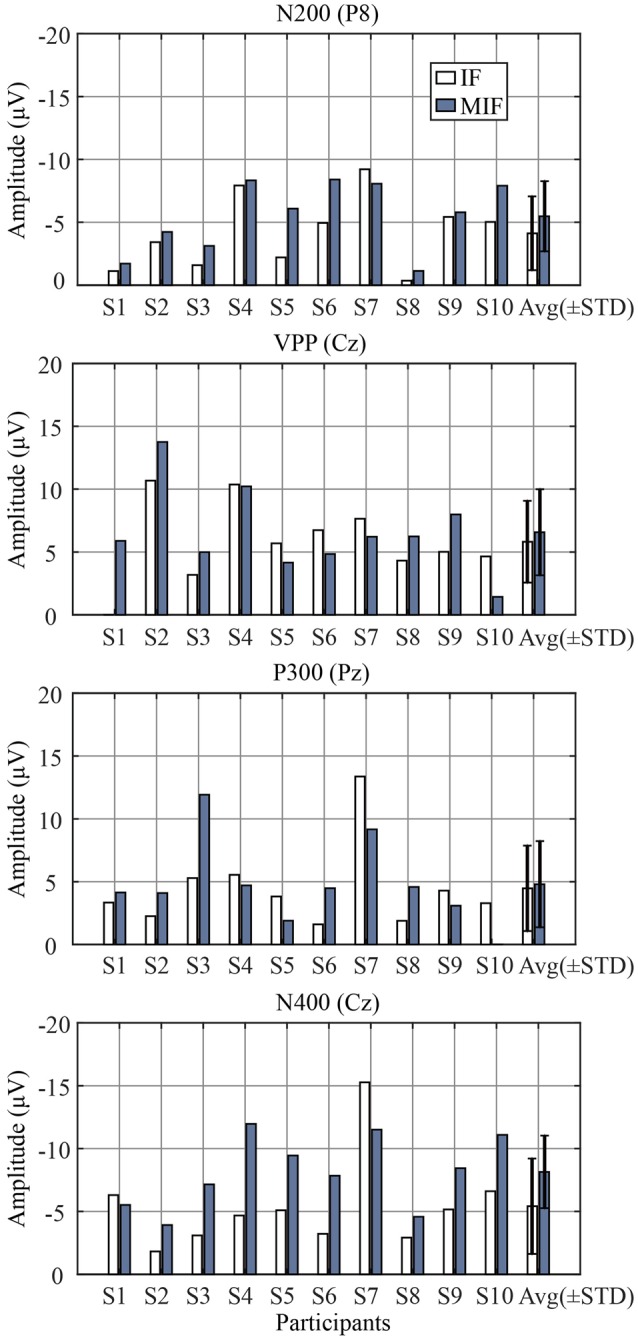
**The amplitudes of N200, VPP, P300, and N400 ERPs per participant**. “STD” denotes the standard deviation.

Figure 4 shows the amplitude of the grand averaged ERP from target flashes across all participants. The baseline was extracted from 100 ms before each deviant stimulus. The N200 ERP on channel P8 (Czigler et al., 2006; Folstein and Van Petten, 2008; Kimura et al., 2010a; Czigler, 2014), the VPP on channel Cz (Jeffreys, 1989), the P300 ERP on channel Pz (He et al., 2013), and the N400 ERP on channel Cz (Duncan et al., 2009) were selected to analyze the effect on the amplitude between two paradigms. The adaptive mean method (Clayson et al., 2013) was used to measure the peak amplitude of the ERPs. The average 25 ms pre-peak to 25 ms post peak amplitudes were extracted around the most positive or negative peaks between 100 and 300 ms (peak negative, N200), 200–350 (peak positive, VPP), 301–500 ms (peak positive, P300), and 401–800 ms (peak negative, N400). The results showed that there were no significant differences between the IF and MIF patterns in VPP (*t* = 0.4269, *P* > 0.05) and P300 (*t* = 0.7578, *P* > 0.05) amplitudes. The amplitudes of the N200 (*t* = 0.0242, *P* < 0.05) and N400 (*t* = 0.0222, *P* < 0.05) of the MIF-pattern were significantly higher than those recorded during the IF-pattern (see Figure 5).

**Figure 6:**
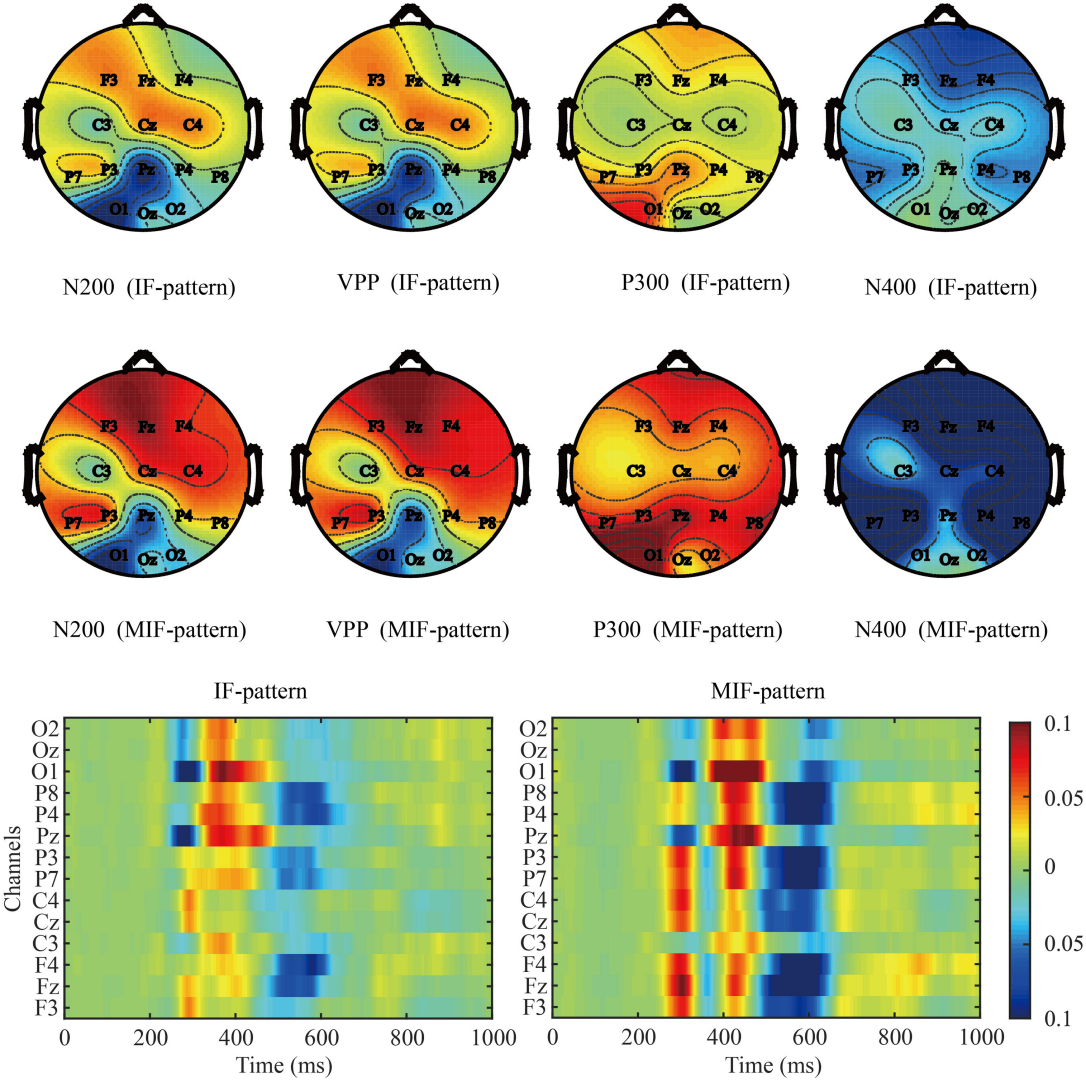
**The ***r***-squared values averaged over participants using offline data**.

Figure 6 shows the grand averaged *r*-squared values of the ERPs and the topographic maps of the N200, VPP, P300, and N400 components.

## Discussions

In this paper, the mismatch inverted face pattern (MIF-pattern) was presented to improve the performance of the inverted face pattern (IF-pattern) based BCIs. The online results showed that the classification accuracies and ITRs achieved with the MIF-pattern were significantly higher than those achieved with the IF-pattern (*p* < 0.05). Two of the participants obtained 100% classification accuracies with both patterns, other participants obtained higher classification accuracies with the MIF-pattern compared to the IF-pattern.

The face patterns evoked large N200 and N400 ERPs. A mismatch negativity (MMN) was elicited when a stimulus was incongruent with the sensory memory trace of a standard stimulus (Näätänen, 1992; Kimura et al., 2010b; Kimura, 2012). The visual MMN was elicited in response to a stimulus that was preceded by four or more repetitions of the other stimuli, while it was not elicited in response to a stimulus that was preceded by one, two, or three repetitions of the other stimuli (Kimura et al., 2006). In order to evoke a stable mismatch negative potential, the trials with < 4 standard stimuli before the deviant stimuli were decreased with the method used in this study (See Section Experiment Setup, Off-, and Online Protocols). Table 1 showed that high classification accuracies could be obtained from these single trials with four or more standard stimuli before the deviant stimuli.

The ERP data showed that a visual MMN was elicited by a stimulus that was incongruent with the sensory memory trace of a standard stimulus which was consistent with the MMN literature (Näätänen, 1992; Kimura et al., 2010b; Kimura, 2012). It was reported that the MMN will not be elicited without establishing a predictive model of the standard stimulus (Squires et al., 1975). In this paper, the presentations of the standard (upright face) stimulus at the target location established a predictive model of the standard stimulus, and the model was disrupted by presentations of the target (inverted face) stimulus. It was shown that N200 amplitude of the MI-F pattern was significantly larger than the N200 of the IF-pattern (*p* < 0.05). In this experiment, we had no a priori reason to expect significantly larger N400s in the MI-F pattern. However, the N400 recorded during the MIF-pattern was significantly larger than during the IF-pattern (*p* < 0.05; see Figure 5). A possible explanation is that the MI-F pattern produced a coherent pattern that provided a clear mismatch of shape, which did not exist in the IF-pattern (Wang et al., 2004; Szűcs et al., 2007).

Zhang et al. designed a BCI based on inverted faces (Zhang et al., 2012). In their study, the averaged online ITR reached 38.7 bits/min, which was higher than that of MIF-pattern (27.78 bits/min). However, the averaged accuracy achieved with the MIF-pattern (99.58%) was a lot higher than the (88.7%) in Zhang's work. It is noteworthy that the condition was different between Zhang's work and this work. For example, the SOA in our study was 300 ms, but it was 180 ms in Zhang's work. Furthermore, more targets (12 targets) were used in this study compared to Zhang's work (8 targets). So the comparison of two studies is not trivial. The vMMN BCI was similar with this work (Jin et al., 2015). The online accuracy in that work was 97.5% and the ITR was 25.1 bit/min. Thus, the performance of that system was lower than our present work and our work has improved the BCI.

## Conclusions

In this paper, a mismatch inverted face (MIF)-pattern was presented and evaluated to assess its effect on the performance of ERP-based BCIs. The results show that the MIF-pattern yielded better performance compared to the inverted face (IF)-pattern in terms of classification accuracy and ITRs. This work also has the potential application. For example, when patients suffering from ALS reach an advanced stage, they lose the ability to move or speak. The BCI speller would help them “talk” to others. So, in our further work, we will verify our study on patients and add more targets to build a speller system.

## Author contributions

SZ did the most of the work on experiment and manuscript. JJ offered the idea of this paper. ID helped to improve the paper. XW helped to improve the experiment. AC did some work on discussion.

## Funding

This work was supported in part by the Grant National Natural Science Foundation of China, under Grant Nos. 61573142, 61203127, 91420302, and 61305028. This work was also supported by the Fundamental Research Funds for the Central Universities (WG1414005, WH1314023, and WH1516018).

### Conflict of interest statement

The authors declare that the research was conducted in the absence of any commercial or financial relationships that could be construed as a potential conflict of interest.

## References

[B1] Bobes M. A. Martín M. Olivares E. Valdés-Sosa M. (2000). Different scalp topography of brain potentials related to expression and identity matching of faces. Cogn. Brain Res. 9, 249–260. 10.1016/S0926-6410(00)00003-3 10808136

[B2] Clayson P. E. Baldwin S. A. Larson M. J. (2013). How does noise affect amplitude and latency measurement of event related potentials (ERPs)? A methodological critique and simulation study. Psychophysiology 50, 174–186. 10.1111/psyp.12001 23216521

[B3] Czigler I. (2014). Visual mismatch negativity and categorization. Brain Topogr. 27, 590–598. 10.1007/s10548-013-0316-8 24057352

[B4] Czigler I. Weisz J. Winkler I. (2006). ERPs and deviance detection: visual mismatch negativity to repeated visual stimuli. Neurosci. Lett. 401, 178–182. 10.1016/j.neulet.2006.03.018 16600495

[B5] Duncan C. C. Barry R. J. Connolly J. F. Fischer C. Michie P. T. Näätänen R.. (2009). Event-related potentials in clinical research: guidelines for eliciting, recording, and quantifying mismatch negativity, P300, and N400. Clin. Neurophysiol. 120, 1883–1908. 10.1016/j.clinph.2009.07.045 19796989

[B6] Farwell L. A. Donchin E. (1988). Talking off the top of your head: toward a mental prosthesis utilizing event-related brain potentials. Electroencephalogr. Clin. Neurophysiol. 70, 510–523. 10.1016/0013-4694(88)90149-6 2461285

[B7] Folstein J. R. Van Petten C. (2008). Influence of cognitive control and mismatch on the N2 component of the ERP: a review. Psychophysiology 45, 152–170. 10.1111/j.1469-8986.2007.00602.x 17850238 PMC2365910

[B8] Gruss L. F. Wieser M. J. Schweinberger S. R. Keil A. (2012). Face-Evoked steady-state visual potentials: effects of presentation rate and face inversion. Front. Hum. Neurosci. 6:316. 10.3389/fnhum.2012.00316 23205009 PMC3506985

[B9] He B. Gao S. Yuan H. Wolpaw J. (2013). Brain–computer interfaces, in Neural Engineering, ed He B. (New York, NY: Springer), 87–151.

[B10] Hoffmann U. Vesin J.-M. Ebrahimi T. Diserens K. (2008). An efficient P300-based brain–computer interface for disabled subjects. J. Neurosci. Methods 167, 115–125. 10.1016/j.jneumeth.2007.03.005 17445904

[B11] Hong B. Guo F. Liu T. Gao X. Gao S. (2009). N200-speller using motion-onset visual response. Clin. Neurophysiol. 120, 1658–1666. 10.1016/j.clinph.2009.06.026 19640783

[B12] Hwang H.-J. Kim S. Choi S. Im C.-H. (2013). EEG-based brain-computer interfaces: a thorough literature survey. Int. J. Hum. Comput. Interact. 29, 814–826. 10.1080/10447318.2013.780869

[B13] Jeffreys D. (1989). A face-responsive potential recorded from the human scalp. Exp. Brain Res. 78, 193–202. 10.1007/BF00230699 2591512

[B14] Jin J. Allison B. Z. Sellers E. W. Brunner C. Horki P. Wang X.. (2011a). An adaptive P300-based control system. J. Neural Eng. 8:036006. 10.1088/1741-2560/8/3/036006 21474877 PMC4429775

[B15] Jin J. Allison B. Z. Sellers E. W. Brunner C. Horki P. Wang X.. (2011b). Optimized stimulus presentation patterns for an event-related potential EEG-based brain–computer interface. Med. Biol. Eng. Comput. 49, 181–191. 10.1007/s11517-010-0689-8 20890671

[B16] Jin J. Allison B. Z. Wang X. Neuper C. (2012). A combined brain–computer interface based on P300 potentials and motion-onset visual evoked potentials. J. Neurosci. Methods 205, 265–276. 10.1016/j.jneumeth.2012.01.004 22269596

[B17] Jin J. Allison B. Z. Zhang Y. Wang X. Cichocki A. (2014a). An erp-based bci using an oddball paradigm with different faces and reduced errors in critical functions. Int. J. Neural Syst. 24:1450027. 10.1142/s0129065714500270 25182191

[B18] Jin J. Daly I. Zhang Y. Wang X. Cichocki A. (2014b). An optimized ERP brain–computer interface based on facial expression changes. J. Neural Eng. 11:036004. 10.1088/1741-2560/11/3/036004 24743165

[B19] Jin J. Sellers E. W. Zhang Y. Daly I. Wang X. Cichocki A. (2013). Whether generic model works for rapid ERP-based BCI calibration. J. Neurosci. Methods 212, 94–99. 10.1016/j.jneumeth.2012.09.020 23032116 PMC3658461

[B20] Jin J. Sellers E. W. Zhou S. Zhang Y. Wang X. Cichocki A. (2015). A P300 brain–computer interface based on a modification of the mismatch negativity paradigm. Int. J. Neural Syst. 25:1550011. 10.1142/S0129065715500112 25804352

[B21] Kaufmann T. Schulz S. Grünzinger C. Kübler A. (2011). Flashing characters with famous faces improves ERP-based brain–computer interface performance. J. Neural. Eng. 8:056016. 10.1088/1741-2560/8/5/056016 21934188

[B22] Kaufmann T. Schulz S. M. Köblitz A. Renner G. Wessig C. Kübler A. (2013). Face stimuli effectively prevent brain–computer interface inefficiency in patients with neurodegenerative disease. Clin. Neurophysiol. 124, 893–900. 10.1016/j.clinph.2012.11.006 23246415

[B23] Kimura M. (2012). Visual mismatch negativity and unintentional temporal-context-based prediction in vision. Int. J. Psychophysiol. 83, 144–155. 10.1016/j.ijpsycho.2011.11.010 22137965

[B24] Kimura M. Katayama J. I. Murohashi H. (2006). Probability-independent and-dependent ERPs reflecting visual change detection. Psychophysiology 43, 180–189. 10.1111/j.1469-8986.2006.00388.x 16712588

[B25] Kimura M. Katayama J. I. Murohashi H. (2008). Attention switching function of memory-comparison-based change detection system in the visual modality. Int. J. Psychophysiol. 67, 101–113. 10.1016/j.ijpsycho.2007.10.009 18031856

[B26] Kimura M. Schröger E. Czigler I. Ohira H. (2010a). Human visual system automatically encodes sequential regularities of discrete events. J. Cogn. Neurosci. 22, 1124–1139. 10.1162/jocn.2009.21299 19583466

[B27] Kimura M. Widmann A. Schröger E. (2010b). Top-down attention affects sequential regularity representation in the human visual system. Int. J. Psychophysiol. 77, 126–134. 10.1016/j.ijpsycho.2010.05.003 20478347

[B28] Long J. Li Y. Wang H. Yu T. Pan J. Li F. (2012). A hybrid brain computer interface to control the direction and speed of a simulated or real wheelchair. IEEE Trans. Neural Syst. Rehabil. Eng. 20, 720–729. 10.1109/TNSRE.2012.2197221 22692936

[B29] Lu S. Guan C. Zhang H. (2009). Unsupervised brain computer interface based on intersubject information and online adaptation. IEEE Trans. Neural Syst. Rehabil. Eng. 17, 135–145. 10.1109/TNSRE.2009.2015197 19228561

[B30] Mak J. Arbel Y. Minett J. McCane L. Yuksel B. Ryan D.. (2011). Optimizing the P300-based brain–computer interface: current status, limitations and future directions. J. Neural Eng. 8:025003. 10.1088/1741-2560/8/2/025003 21436525

[B31] Martens S. Hill N. Farquhar J. Schölkopf B. (2009). Overlap and refractory effects in a brain–computer interface speller based on the visual P300 event-related potential. J. Neural Eng. 6:026003. 10.1088/1741-2560/6/2/026003 19255462

[B32] Näätänen R. (1992). Attention and Brain Function. New Jersey, NJ: Psychology Press.

[B33] Penney T. B. Mecklinger A. Nessler D. (2001). Repetition related ERP effects in a visual object target detection task. Cogn. Brain. Res. 10, 239–250. 10.1016/S0926-6410(00)00041-0 11167048

[B34] Sellers E. W. Krusienski D. J. McFarland D. J. Vaughan T. M. Wolpaw J. R. (2006). A P300 event-related potential brain–computer interface (BCI): the effects of matrix size and inter stimulus interval on performance. Biol. Psychol. 73, 242–252. 10.1016/j.biopsycho.2006.04.007 16860920

[B35] Squires N. K. Squires K. C. Hillyard S. A. (1975). Two varieties of long-latency positive waves evoked by unpredictable auditory stimuli in man. Electroencephalogr. Clin. Neurophysiol. 38, 387–401. 10.1016/0013-4694(75)90263-1 46819

[B36] Szűcs D. Soltész F. Czigler I. Csépe V. (2007). Electroencephalography effects to semantic and non-semantic mismatch in properties of visually presented single-characters: the N2b and the N400. Neurosci. Lett. 412, 18–23. 10.1016/j.neulet.2006.08.090 17141414

[B37] Tobias K. Völker S. Gunesch L. Kübler A. (2013). Spelling is just a click away–a user-centered brain-computer interface including auto-calibration and predictive text entry. Front. Neurosci. 6:72. 10.3389/fnins.2012.00072 22833713 PMC3400942

[B38] Townsend G. Shanahan J. Ryan D. B. Sellers E. W. (2012). A general P300 brain–computer interface presentation paradigm based on performance guided constraints. Neurosci. Lett. 531, 63–68. 10.1016/j.neulet.2012.08.041 22960261 PMC3646331

[B39] Wang Y. Cui L. Wang H. Tian S. Zhang X. (2004). The sequential processing of visual feature conjunction mismatches in the human brain. Psychophysiology 41, 21–29. 10.1111/j.1469-8986.2003.00134.x 14692997

[B40] Wolpaw J. R. Birbaumer N. Heetderks W. J. McFarland D. J. Peckham P. H. Schalk G.. (2000a). Brain-computer interface technology: a review of the first international meeting. IEEE Trans. Rehabil. Eng. 8, 164–173. 10.1109/TRE.2000.847807 10896178

[B41] Wolpaw J. R. Birbaumer N. McFarland D. J. Pfurtscheller G. Vaughan T. M. (2002). Brain–computer interfaces for communication and control. Clin. Neurophysiol. 113, 767–791. 10.1016/S1388-2457(02)00057-3 12048038

[B42] Wolpaw J. R. McFarland D. J. Vaughan T. M. (2000b). Brain-computer interface research at the Wadsworth Center. IEEE Trans. Rehabil. Eng. 8, 222–226. 10.1109/86.847823 10896194

[B43] Yin E. Zeyl T. Saab R. Chau T. Hu D. Zhou Z. (2015). A hybrid brain-computer interface based on the fusion of P300 and SSVEP scores. IEEE Trans. Neural Syst. Rehabil. Eng. 23, 693–701. 10.1109/TNSRE.2015.2403270 25706721

[B44] Yin E. Zhou Z. Jiang J. Chen F. Liu Y. Hu D. (2013). A novel hybrid BCI speller based on the incorporation of SSVEP into the P300 paradigm. J. Neural. Eng. 10:026012. 10.1088/1741-2560/10/2/026012 23429035

[B45] Yin E. Zhou Z. Jiang J. Chen F. Liu Y. Hu D. (2014). A speedy hybrid BCI spelling approach combining P300 and SSVEP. IEEE Trans. Biomed. Eng. 61, 473–483. 10.1109/TBME.2013.2281976 24058009

[B46] Zhang D. Song H. Xu R. Zhou W. Ling Z. Hong B. (2013). Toward a minimally invasive brain–computer interface using a single subdural channel: a visual speller study. Neuroimage 71, 30–41. 10.1016/j.neuroimage.2012.12.069 23313779

[B47] Zhang Y. Zhao Q. Jin J. Wang X. Cichocki A. (2012). A novel BCI based on ERP components sensitive to configural processing of human faces. J. Neural Eng. 9:026018. 10.1088/1741-2560/9/2/026018 22414683

[B48] Zhang Y. Zhou G. Zhao Q. Jin J. Wang X. Cichocki A. (2013). Spatial-temporal discriminant analysis for ERP-based brain-computer interface. IEEE Trans. Neural Syst. Rehabil. Eng. 21, 233–243. 10.1109/TNSRE.2013.2243471 23476005

